# Supplementary material for: A Humanized Yeast Phenomic Model of Deoxycytidine Kinase to Predict Genetic Buffering of Nucleoside Analog Cytotoxicity
Source: Genes (Basel). 2019 Sep 30;10(10):770. doi: 10.3390/genes10100770 (PMC6826991; doi:10.3390/genes10100770)
Supplement: Supplementary file 1 [file genes-10-00770-s001.zip › Additional_Files/Additional_File7_GOTermHeatmaps.zip!/Additional_File7_GOTermHeatmaps/N-terminal_protein_amino_acid_acetylation.pdf]

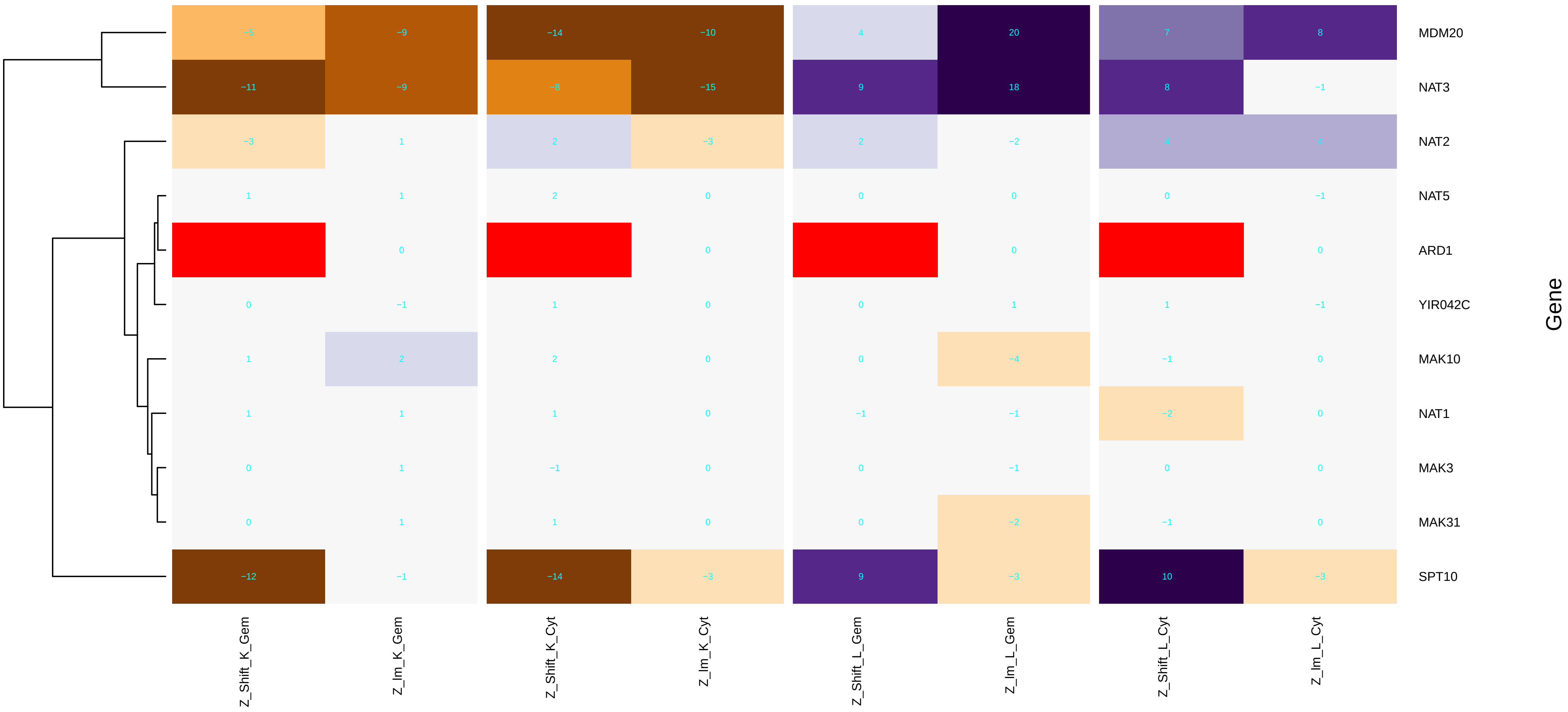
Z_Shift_K_Gem
Z_Im_K_Gem
Z_Shift_K_Cyt
Z_Im_K_Cyt
Z_Shift_L_Gem
Z_Im_L_Gem
Z_Shift_L_Cyt
Z_Im_L_Cyt
MDM20: −5, −9, −14, −10, 4, 20, 7, 8
NAT3: −11, −9, −8, −15, 9, 18, 8, −1
NAT2: −3, 1, 2, −3, 2, −2, 4, 4
NAT5: 1, 1, 2, 0, 0, 0, 0, −1
ARD1: 0, 0, 0, 0
YIR042C: 0, −1, 1, 0, 0, 1, 1, −1
MAK10: 1, 2, 2, 0, 0, −4, −1, 0
NAT1: 1, 1, 1, 0, −1, −1, −2, 0
MAK3: 0, 1, −1, 0, 0, −1, 0, 0
MAK31: 0, 1, 1, 0, 0, −2, −1, 0
SPT10: −12, −1, −14, −3, 9, −3, 10, −3
Gene
